# The placebo effect in the motor domain is differently modulated by the external and internal focus of attention

**DOI:** 10.1038/s41598-018-30228-9

**Published:** 2018-08-16

**Authors:** Giacomo Rossettini, Mehran Emadi Andani, Francesco Dalla Negra, Marco Testa, Michele Tinazzi, Mirta Fiorio

**Affiliations:** 1 Department of Neurosciences, Biomedicine and Movement Sciences, University of Verona, Verona, Italy; 2 Department of Neuroscience, Rehabilitation, Ophthalmology, Genetics, Maternal and Child Health, University of Genoa, Campus of Savona, Genoa, Italy

## Abstract

Among the cognitive strategies that can facilitate motor performance in sport and physical practice, a prominent role is played by the direction of the focus of attention and the placebo effect. Consistent evidence converges in indicating that these two cognitive functions can influence the motor outcome, although no study up-to-now tried to study them together in the motor domain. In this explorative study, we combine for the first time these approaches, by applying a placebo procedure to increase force and by manipulating the focus of attention with explicit verbal instructions. Sixty healthy volunteers were asked to perform abduction movements with the index finger as strongly as possible against a piston and attention could be directed either toward the movements of the finger (internal focus, IF) or toward the movements of the piston (external focus, EF). Participants were randomized in 4 groups: two groups underwent a placebo procedure (Placebo-IF and Placebo-EF), in which an inert treatment was applied on the finger with verbal information on its positive effects on force; two groups underwent a control procedure (Control-IF and Control-EF), in which the same treatment was applied with overt information about its inefficacy. The placebo groups were conditioned about the effects of the treatment with a surreptitious amplification of a visual feedback signalling the level of force. During the whole procedure, we recorded actual force, subjective variables and electromyography from the hand muscles. The Placebo-IF group had higher force levels after the procedure than before, whereas the Placebo-EF group had a decrease of force. Electromyography showed that the Placebo-IF group increased the muscle units recruitment without changing the firing rate. These findings show for the first time that the placebo effect in motor performance can be influenced by the subject’s attentional focus, being enhanced with the internal focus of attention.

## Introduction

Motor performance can be enhanced in athletes and non-athletes by mean of placebo procedures^1–6^, in which inert treatments or substances are administered together with verbal information about their powerful effects on the motor outcome. Strong placebo effects have been described in several sport disciplines, such as cycling^7,8^, running^9^, sprint^10^ and weight lifting^11,12^. For instance, well-trained cyclists, who thought to have ingested caffeine, showed improved performance, even if they received a placebo^8^. Placebo procedures work even in non-athletes, as shown with different motor tasks, such as leg-extension^13^, simulation of a sport competition^14^, finger movements^15,16^, arm movements^17^. More recently, the neurophysiological correlates of these effects have been uncovered by applying transcranial magnetic stimulation over the primary motor cortex of healthy non-athletes engaged in a force task^16^. This study showed that after a placebo procedure there was an increased excitability of the corticospinal system, as demonstrated by the enhanced amplitude of the motor evoked potentials and reduced duration of the cortical silent period^16^. Hence, the placebo procedure seems to boost the activity of the motor system and this could in turn favour motor performance.

All this evidence suggests that placebo procedures can be exploited as cognitive strategies, in addition to physical training, to boost motor performance. However, it is still unknown what type of verbal instruction is more suitable to induce a robust placebo effect in the motor domain. Previous research in pain has shown that when participants receive verbal instructions to focus on the body part in which the effects of a treatment are expected, the placebo response is enhanced^18^. This suggests that different verbal information can direct attention towards or away from the body and this has an impact on the placebo response. The assumption here is that the expectation induced through the placebo procedure can influence cognitive processing and behavior only if it biases attention toward the relevant somatic information^18^. Concerning the placebo effect in the motor domain, no study up-to-now has tackled this issue, despite the general evidence showing that the direction of attention has a strong influence on motor performance. Specifically, plenty of literature suggests that verbal instructions to focus attention away from the body movements (the so-called, external focus of attention, EF) lead to better motor performance as compared to verbal instructions to focus on the body movements (internal focus of attention, IF)^19,20^. This pattern seems to be associated to changes at the neuromuscular level, with a generally lower EMG activity and fewer co-contraction of agonist and antagonist muscles when the movements are executed with an external focus of attention^21,22^. Based on this evidence, it was suggested that focusing attention away from the movements of the body may result in a more efficient and economical muscular activation^20^. However, some studies have questioned the absolute advantage of the external over the internal focus of attention, suggesting that the external focus is not always the best strategy for all the subjects^23–26^. Different individual factors can influence the effect of the attentional focus instructions on the motor outcome. For instance individuals with a strong disposition to the conscious control of movements benefit more from the internal than from the external focus of attention^27^. Furthermore, subjects differ for task-specific preferences toward the internal or the external attentional focus, presenting with better performance only when the preferred focus is adopted^28–32^.

The question is whether the placebo effect in motor performance is differently modulated by the external or internal focus of attention and whether these modulations are associated to different neuromuscular activation. To answer this question, we applied a placebo procedure to induce an increase of force production in healthy volunteers^16,33^ and manipulated participants’ focus of attention with explicit verbal instructions. More precisely, participants had to perform abduction movements of the index finger against a piston and attention could be directed either toward the movements of the finger (IF) or toward the movements of the piston (EF). During the whole procedure, we recorded electromyography from the muscle involved in the task (the first dorsal interosseous) and from a control muscle (abductor digiti minimi). In this way, we could uncover any fine-tuned changes in the neuromuscular pattern of activation related to the different conditions. The general assumption is that a more parsimonious pattern of muscular activation is indicative of more efficient and automatic movement execution that leads in turn to a good motor performance with less effort and fatigue^34^.

## Materials and Methods

### Participants

A total of 60 healthy subjects (25 women; mean age, 23.2 ± 3.3 years) were recruited, using a convenience sampling, from the student population of the University of Verona and were divided into four groups: 15 subjects (7 women; mean age, 22.5 ± 3.2 years) entered the Placebo Internal focus of attention group (Placebo-IF), 15 subjects (5 women; mean age, 22.3 ± 2.4 years) entered the Placebo External focus of attention group (Placebo-EF), 15 subjects (7 women; mean age, 23.9 ± 3.9 years) entered the Control Internal focus of attention group (Control-IF), 15 subjects (6 women; mean age, 24.2 ± 3.5 years) entered the Control External focus of attention (Control-EF).

Computation of the sample size is reported in the Supplementary information.

All the subjects subscribed an informed consent form in which the experimental procedure was explained in detail, before participation to the study. They were naive to the real aims of the study that were discussed only at the end of the experimental procedure. The study was conducted according to the principles expressed in the Declaration of Helsinki and was approved by the ethical committee of the Department of Neuroscience, Biomedicine and Movement Sciences, University of Verona, Italy. None of the participants presented neurological, psychiatric, or other medical problems.

### Motor task

The measurements of force production were acquired using a motor task similar to our previous studies^16,33^. The research was conducted in a quiet room. Healthy volunteers were seated in a comfortable chair with elbows semi-flexed and forearms pronated, fully relaxed, and supported by the arm of the chair. Subjects performed abduction movements of the right index finger to press a piston connected to a force transducer (DS BC302). This movement is not executed in daily life and it can be effectively controlled in an experimental setting, limiting the influence of external variables (e.g., practice). A custom-made device was used to restrain the hand during the motor task. This strategy permitted to isolate the first dorsal interosseous (FDI) muscle during the task. The tip of the piston was positioned at 80% length of the index finger, over the distal interphalangeal joint.

**Figure 1:**
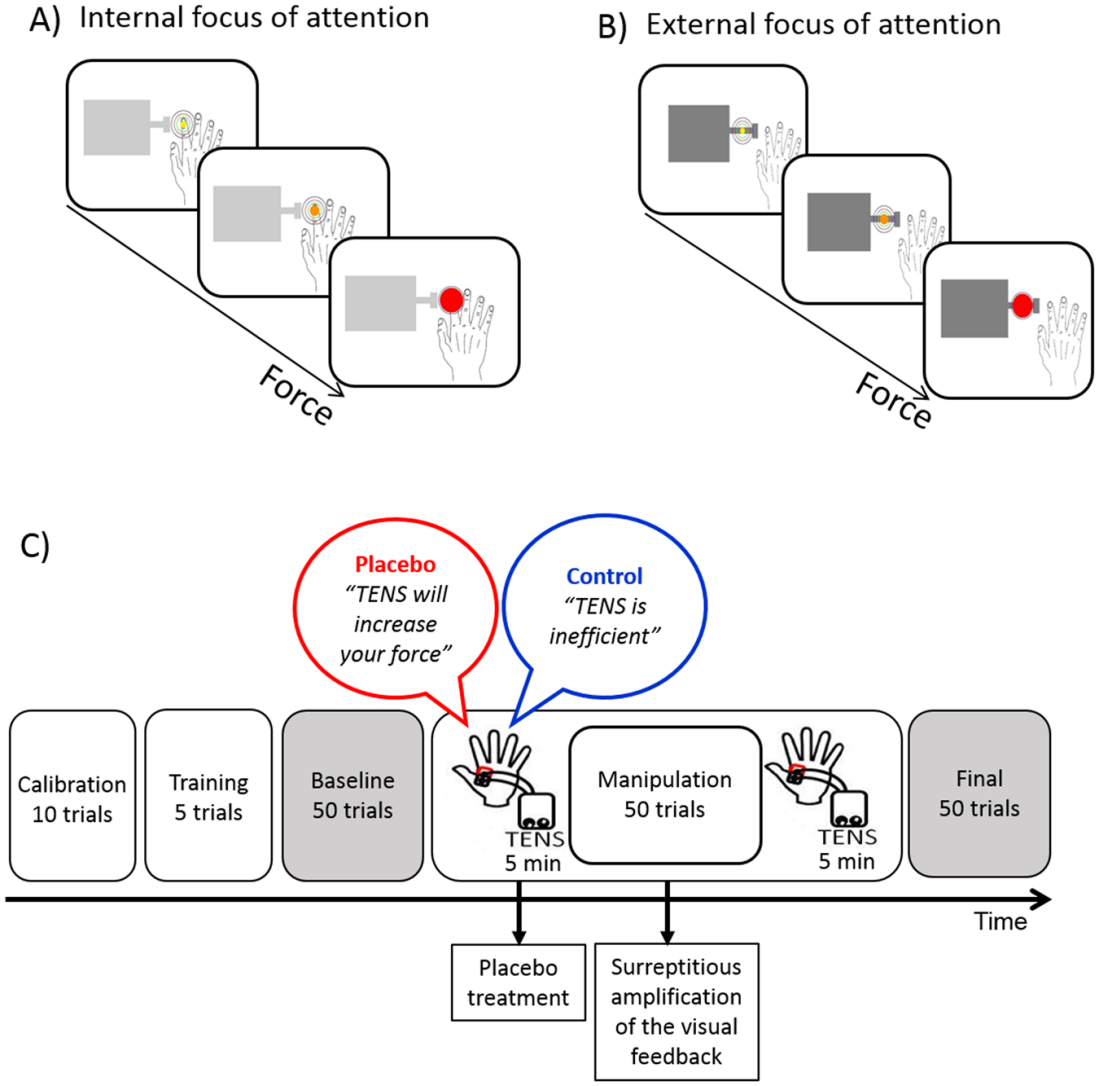
Schematic representation of the motor task and of the procedure. Participants were asked to press a piston connected to a force transducer, as strongly as possible, by performing abduction movements with the right index finger. A visual feedback on a PC monitor signalled the level of force. The feedback consisted of a circular cursor that and changed in dimension and colour depending on the amount of force. The context surrounding the cursor was defined according to the direction of the focus of attention. In the internal focus of attention (**A**), participants received instructions to focus their attention on the movements of the finger and, consequently, the cursor was located on the visual representation of the finger. In the external focus of attention (**B**), participants received instructions to focus their attention on the movements of the piston and, consequently, the cursor was located on the piston. In the two cases, the absolute position of the cursor on the screen was identical. The procedure (**C**) consisted of three sessions: the baseline and final session were identical and allowed us to compare performance before and after the placebo procedure that consisted of verbal suggestion (different for the placebo and control groups) and of conditioning specifically applied to the placebo groups. TENS was used as placebo treatment. Before starting the procedure, the maximum voluntary force (MVF) of each participant was measured in an initial calibration phase (10 trials). Moreover, participants trained with the motor task by performing 5 trials.

Participants were told to press the piston as strongly as possible and received a visual feedback on the amount of force produced. More precisely, finger pressures against the piston were real-time linearly converted into colour and dimension changes of a circular cursor visible on a 25.5 × 41 cm PC monitor. The stronger the force exerted on the piston, the greater and redder the cursor appeared on the PC monitor (Fig. 1A,B). In this way, the size and colour of cursor served as a visual feedback for the subject about the amount of applied force. Cursor’s changes were obtained using the following formula:

$${\rm{d}}={\rm{k}}\times {\rm{F}}$$

where k is a constant converting coefficient and F is the force (in Newton, N) applied to the piston.

To rule out any effect of spatial attention, the absolute position of the cursor on the screen was the same for all the participants, i.e., 8.5 cm from the upper border and 20.5 cm from the right border of the PC monitor (Fig. 1A,B). However, depending on the focus of attention (internal or external, see below for more details) the relative location of the cursor varied within a surrounding context. The context consisted in a graphical representation of the piston and of the participant’s hand. In case of the internal focus of attention, the cursor was located on the representation of the index finger (Fig. 1A), whereas in case of the external focus of attention it was located on the representation of the piston (Fig. 1B). In this way, the visual feedback was congruent with the direction of the focus of attention.

Before starting the experimental session, the maximum voluntary force (MVF) was measured by asking each participant to perform 10 pressures at their maximal force output. The peak force amplitude averaged across the 10 trials was considered as the subject’s MVF. During the following experimental sessions, this value was used to scale the circular changes of the cursor. Namely, the central circle was surrounded by three borders that corresponded to 80%, 100% and 120% MVF (Fig. 1A,B). This calibration procedure prevented force saturation during the execution of the task and permitted for a margin of force improvement during the experiment. After the calibration phase and before starting the main experiment, the subjects performed a short training of 5 trials to familiarize with the task.

At the beginning of each trial, a “START” signal appeared at the centre of the PC monitor and participants were asked to press the mouse with the left hand to initiate the trial. After having pressed the mouse, the cursor with the surrounding context appeared on the monitor and the participant could start pressing the piston. Each trial lasted 1100 ms and subjects were required to maintain the achieved pressure until the end of the trial. The task entailed 50 trials and was repeated in three consecutive sessions (baseline, manipulation and final, see details below) executed within 1 day.

### Procedure

At the beginning of the experimental procedure, subjects received different verbal instructions for the two types of attentional focus, as typically done to direct the focus of attention^35^. Participants with the internal focus of attention (IF) were told: “*During the motor task, please focus on the movements of your finger. The visual feedback on the screen represents the force of your finger*”. Participants with the external focus of attention (EF) were told: “*During the motor task, please focus on the movements of piston. The visual feedback on the screen represents the force exerted on the piston*”. These instructions were reminded at the beginning of each experimental session and during the motor task. The ability to maintain the focus of attention was assessed at the end of the experiment by asking participants to estimate the duration of their focus of attention on the finger’s movements (IF) or on the piston’s movements (EF), depending on the groups, by means of a 10-cm-long visual analogue scale (VAS), ranging from 0 (never) to 10 (always)^36^.

A protocol consisting of three sessions (baseline, manipulation, final) was adopted (Fig. 1C). In the baseline session, participants executed the motor task (50 trials). This session allowed to measure the subjects’ initial performance. The final session consisted again in the similar execution of the motor task (50 trials) and allowed to measure the subjects’ final performance, after the experimental manipulation.

During the manipulation, two placebo groups (one with internal focus of attention, Placebo-IF, and one with external focus of attention, Placebo-EF) were tested using a placebo procedure made of a combination of verbal suggestion and conditioning^37^. As placebo treatment, we applied transcutaneous electrical nerve stimulation (TENS) for 5 min over the hand^16,33^. TENS was applied to the FDI muscle using an electro-stimulator with a pair of self-adhesive electrodes (1.5 cm diameter). The negative electrode was located over the FDI muscle and the positive electrode was placed 3 cm distally near the muscle. The current was an asymmetric rectangular biphasic waveform with a frequency of 10 Hz and a pulse width of 150 μs. The intensity was adjusted so that the subjects vaguely perceived the stimulation without muscle contraction. To create expectation of good motor performance, TENS was directly applied over the region of the hand muscle involved in the task and caused a cutaneous sensation felt by the subject. Moreover, the researcher adopted verbal suggestion to induce expectation about the effects of TENS: “*This is a new treatment used also in the clinical practice that has a direct effect in enhancing force production*”.

To condition the participants on the effects of TENS, a programmed, covert amplification of the cursor’s displacement was introduced progressively. After TENS, unbeknown to the subjects, the changes of the cursor were gradually intensified trial by trial, with an amplification coefficient (α). In particular, amplified size (d′) of the cursor on the monitor was calculated as:

$${\rm{d}}^{\prime} ={\rm{\alpha }}\times {\rm{k}}\times {\rm{F}}$$

where α was the amplification coefficient and increased gradually in steps of 0.0059 at each trial, starting from 1 (i.e., no amplification in the first trial) to a maximum of 1.2 at trial 35, and remained stable until the end of the session (from trial 36 to trial 50). In this way, by applying the same amount of force as in the baseline, the subjects of the placebo groups could see the cursor growing up more than before and therefore believed to be stronger because of the treatment. Amplification was programmed gradually to prevent that subjects became aware of the manipulation. Before starting the final session, TENS was applied again together with verbal suggestion of better motor performance. Subjects then repeated the motor task, but this time without amplification of the cursor’s dimension.

Two control groups (one with internal focus of attention, Control-IF, and one with external focus of attention, Control-EF) performed the same motor task and underwent the TENS treatment as described previously, but with different verbal information and without amplification of the cursor’s dimension. More precisely, these subjects were openly told that they have been assigned to a control group in which the frequency of TENS was completely inefficient in influencing force production. This group performed the motor task in the same way as in the baseline (i.e., without any amplification of the cursor’s dimension). After the application of TENS, before executing the motor task, all the participants were asked to rate whether they expected a change in performance due to TENS and in which direction the change would be, by considering the baseline session as a reference point. This judgment was made on a 7-points number rating scale (NRS), ranging from -3 (“*I expect that my performance will be much worse than at baseline*”) to 3 (“*I expect that my performance will be much better than at baseline*”) with 0 in the middle (“*I expect that my performance will be the same as in the baseline*”)^16,33^. This rating was made twice, once after each TENS application and allowed to check whether the two placebo groups had positive expectations about the effects of the treatment. Soon after the execution of the motor task in the manipulation and final sessions, participants were also asked to judge whether TENS was effective in influencing force, using a 10-cm-long VAS ranging from 0 (“*TENS was not effective at all*”) to 10 (“*TENS was extremely effective*”)^16,33^. This rating allowed us to check whether participants perceived any effect of the treatment on their performance.

### Behavioural data

The mean value of the peak force amplitude obtained from the 50 trials of each session (Force_peak_) was normalized to the maximum force expressed during the initial calibration procedure (MVF). This index was defined with the following formula:

$${\rm{Normalized}}\,{{\rm{Force}}}_{{\rm{peak}}}={{\rm{Force}}}_{{\rm{peak}}}/{\rm{MVF}}\times 100 \% $$

This index gives information on whether the mean force level achieved by the subjects in the different experimental sessions changed in relation to the maximum voluntary force.

The percentage of strong pressures in each session was defined with the following formula:

$${\rm{Percentage}}\,{\rm{of}}\,{\rm{strong}}\,{\rm{pressures}}={{\rm{N}}}_{{\rm{Strong}}{\rm{trials}}}/{{\rm{N}}}_{{\rm{Tot}}{\rm{trials}}}\times 100 \% $$

where N_Tot trials_ is the total number of trials in each session (i.e., 50) and N_Strong trials_ is the number of trials in which the peak force amplitude was above the mean value computed in the baseline. In this way, whereas the first parameter (Normalized Force_peak_) is a measure of the subject’s mean force level achieved in each session in relation to the MVF, the second parameter (percentage of strong pressures) allowed to compute how many times the subject pressed the piston above a certain value, thus giving a measure of the consistency of behavior.

### Subjective data

Soon after the execution of the motor task, participants were asked to judge how strong they have felt during the execution of the task by means of a 10-cm-long VAS from 0 (very weak) to 10 (very strong)^16,33^. They had also to judge the perception of effort on the Borg scale, ranging from 6 (rest) to 20 (maximal effort)^38^.

### EMG recording and data

In each trial, EMG recording began 100 ms before starting the task and finished 100 ms after finishing the task. Surface EMG recordings were obtained from the motor point of the first dorsal interosseous (FDI) and abductor digiti minimi (ADM) muscles of the right hand with bipolar self-adhesive Ag-AgCl electrodes (1.5 × 2.5 cm) in a belly–tendon montage. The ground electrode was attached to the wrist. EMG signals were bandpass filtered (20 Hz to 2.5 kHz; plus 50 Hz notch) (D360, Digitimer), amplified at a gain of 1000 (Digitimer), digitized at 5 kHz with laboratory interface (Cambridge Electronic Design 1401) controlled by Spike 2 (version 6, Cambridge Electronic Design) and analyzed off-line.

After applying 20–500 Hz band pass filter on EMG data, we computed two different indexes to evaluate EMG activity: root mean square (RMS) and zero crossing rate (ZCR)^39,40^.

The RMS was calculated as the root mean square of EMG signal during task performance. This index gives information about the level of EMG activation.

The ZCR index was calculated using the following formula (1):

1 $$ZCR=\frac{1}{N-1}\sum _{i=2}^{N}I({S}_{i}{S}_{i-1} < 0)\,where\,I(A)=\{\begin{array}{c}1\,if\,A\,is\,true\\ 0\,otherwise\end{array}$$

where *S*_*i*_ is *i*^*th*^ sample of EMG signal. In order to increase the signal to noise ratio, before calculating ZCR we considered a threshold of 100 μV and consequently we removed from the EMG the signals with an absolute level below 100 μV. This index gives information about the motor unit recruitment. Higher rate of motor unit recruitment results in higher values of the ZCR^41^. More precisely, an increase in ZCR indicates an increase in the neural drive to the muscles, due to enhanced asynchrony in the firing rates of different muscle fibers^41^. An increase of neural input can be considered as a strategy to improve performance, whereas a reduction of neural input can be a sign of central fatigue^42^. The mean value of each index obtained from the 50 trials of each session was considered as the representative for the session and was entered in the statistical analyses.

### Data handling

Before calculating the mean value of Force_peak_ in each session and subject, behavioral data were screened in two ways. First, trials in which participants did not press the piston after having pressed the mouse key were removed (Placebo-IF: 0%; Placebo-EF: 0.07%; Control-IF: 0%; Control-EF: 0.2%). Second, data of each subject in each session were inspected to exclude potential outliers, i.e., values 2 × SD above or below the mean value (Placebo-IF: 3.73%; Placebo-EF: 4.53%; Control-IF: 4%; Control-EF: 4.4%).

### Statistical analysis

Analyses were executed using SPSS Statistics 21 software (IBM SPSS Statistics 21). Normality of data distribution was checked with Shapiro-Wilk test and the Box-Cox transformation was applied if normality was violated^43^. Data were further inspected to find potential outliers, defined as those subjects whose value in each variable and session was above or below the mean value of the group by 2 times the standard deviation of the group (see Supplementary Table S1).

A preliminary analysis of variance (one-way ANOVA) was performed to check whether the four groups (Placebo-EF, Placebo-IF, Control-EF, Control-IF) were comparable for age, MVF, duration of the focus of attention, expectation about TENS and perception of TENS efficacy.

Behavioural, subjective and EMG data were analysed by means of mixed ANOVA with Session (baseline and final) as within-subjects factor and Procedure (placebo and control) and Focus of attention (internal and external) as between-subjects factors. Post-hoc comparisons were performed by means of one-way ANOVA with the factor Group (Placebo-EF, Placebo-IF, Control-EF, Control-IF) and by means of t-tests for independent or paired samples. Bonferroni correction for multiple comparisons was applied where necessary. The level of significance was set at p < 0.050.

Behavioural data of the manipulation session were further analysed to check whether the four groups had the same amount of force. Since this session was not the same in all the groups (i.e., the placebo groups performed the motor task with a conditioning procedure, whereas the control groups performed the motor task without conditioning), data were analysed separately (see Supplementary information and Table S2). Sensitivity analysis including outliers is reported in Supplementary Table S3.

## Results

**Figure 2:**
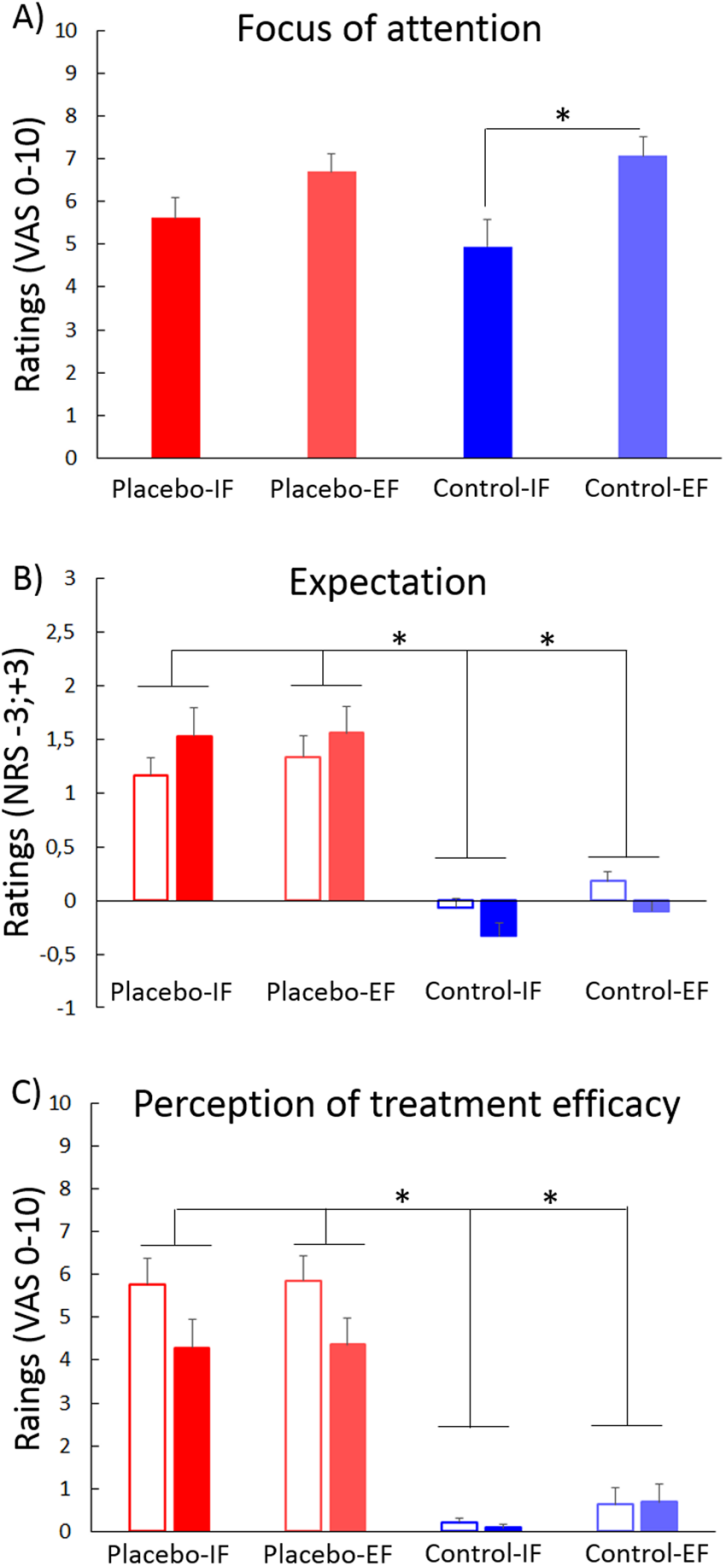
Ratings of focus of attention, expectation and treatment efficacy. All the groups were able to maintain the focus of attention toward the required object (**A**) and the Control-IF group (dark blue bars) declared shorter duration of the focus than the Control-EF group (light blue bars). Expectation of improvement was higher in the two placebo groups (red bars) than in the two control groups (blue bars), both in the first (white bars) and second application (full coloured bars) (**B**). The treatment was perceived as more effective in the two placebo groups (red bars) than in the two control groups (blue bars), both in the first (white bars) and second application (full coloured bars) (**C**). Values are expressed as mean ± SE. *Significant values, p < 0.050.

The four groups did not statistically differ for age (F(3,56) = 1.357, p = 0.265) and MVF (F(3,56) = 0.651, p = 0.586). Moreover, they were all able to maintain the focus of attention toward the direction given by the experimenter, since their VAS score were all different from 0 (t-test for one sample, for all groups p < 0.001), although the ability to keep the focus was different between groups (F(3,56) = 3.95, p = 0.013), as the Control-IF group (4.95 ± 0.62) reported a shorter duration than the Control-EF group (7.07 ± 0.43,p = 0.021) (Fig. 2A). No difference was found between the Placebo-IF (5.61 ± 0.47) and the Placebo-EF group (6.7 ± 0.42, p = 0.729). Expectation was higher in the two placebo groups compared to the two control groups both for the first application (F(3,56) = 25.3, p < 0.001) and for the second application (F(3,56) = 26.7, p < 0.001), suggesting that the procedure was suitable to induce positive expectation in the placebo groups (Fig. 2B). Perception of treatment efficacy was higher in the two placebo groups compared to the two control groups both for the first application (F(3,56) = 43.65, p < 0.001) and for the second application (F(3,56) = 20.17, p < 0.001), hinting again to the suitability of the paradigm to induce positive belief (Fig. 2C).

### Behavioural data

**Figure 3:**
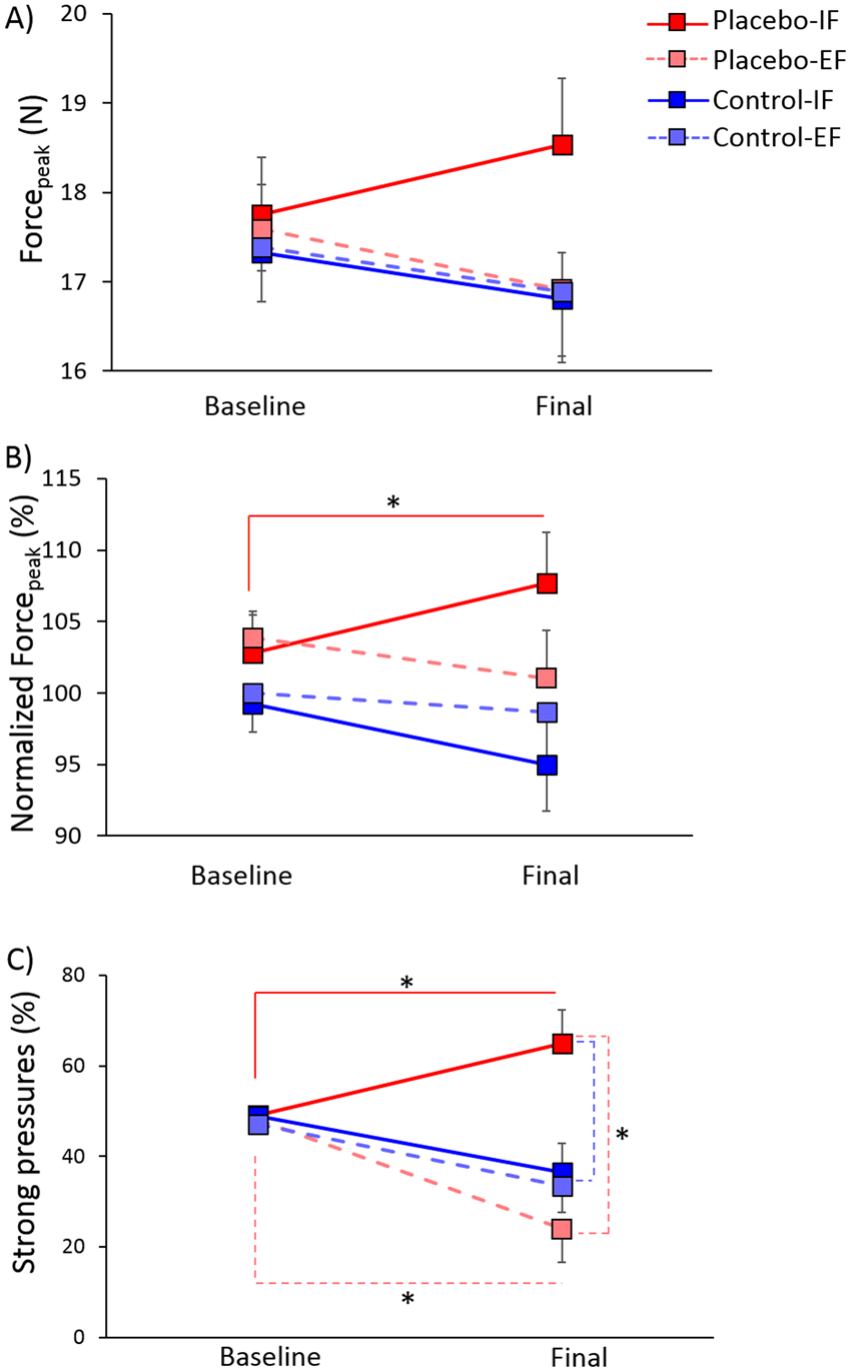
Mean values ± SEM of the behavioural variables. Levels of raw Force_peak_ (**A**), Normalized Force_peak_ (**B**) and percentage of strong pressures (**C**) in the placebo groups (red squares) and in the control groups (blue squares), with internal focus of attention (IF, dark coloured squares) and with external focus of attention (EF, light coloured squares), in the baseline and final sessions. The Placebo-IF group showed a significant increase in performance from the baseline to the final session in the Normalized Force_peak_ and the percentage of strong pressures. A decrease of percentage of strong pressures was observed in the Placebo-EF group. Moreover, a difference was found between the Placebo-IF and the Placebo-EF and the Control-EF groups in the final session. *Significant values, p < 0.050.

Being not normally distributed (Shapiro-Wilk, p < 0.050), behavioural data were submitted to the Box-Cox transformation^43^ before entering into the analysis. Analysis of Force_peak_ did not reveal any significant effect (for all the factors and interactions, p > 0.102) (Fig. 3A).

Analysis of Normalized Force_peak_ disclosed a significant effect of Procedure (F(1,52) = 4.94, p = 0.031), due to higher values in the placebo procedure (mean ± SEM, 103.85 ± 1.8%) compared to the control procedure (98.22 ± 1.8%). The interaction Session × Procedure × Focus of attention was also significant (F(1,52) = 4.75, p = 0.034). Post-hoc comparisons showed that in the Placebo-IF group there was an increase of Normalized Force_peak_ in the final session (107.7 ± 3.42%) compared to baseline (102.79 ± 2.12%) (p = 0.015) (Fig. 3B). Conversely, the other three groups had a stable performance across sessions (Placebo-EF: p = 0.381, Control-IF: p = 0.131, Control-EF: p = 0.807). One-way ANOVA with the factor Group disclosed no significant difference between groups neither in the baseline (p = 0.394) nor in the final session (p = 0.097).

Analysis of the percentage of strong pressures revealed a significant effect of the Focus of attention (F(1,50) = 6.5, p = 0.009), due to higher values with the internal focus (49.85 ± 2.91%) compared to the external focus (36.31 ± 3.01%). The interaction Procedure × Focus of attention was also significant (F(1,50) = 5.77, p = 0.020). Post-hoc comparisons showed that the Placebo-IF group had in general higher percentage of strong pressures (57.0 ± 3.96%) compared to the Placebo-EF: (36.31 ± 4.26%, p = 0.003) and to the Control-EF group (40.31 ± 4.26%, p = 0.033). The interaction Session × Focus of attention was also significant (F(1,50) = 5.81, p = 0.020). Post-hoc comparisons showed that in the final session the percentage of strong pressures was higher with the internal (50.7 ± 5.74%) compared to the external focus of attention (28.77 ± 5.94%, p = 0.012). Moreover, with the internal focus of attention the percentage of strong pressures was higher in the final session compared to baseline (49 ± 0.83%, p = 0.040), whereas no difference was found between sessions in the external focus of attention (p = 0.235). More interestingly, the interaction Session × Procedure × Focus of attention was significant (F(1,50) = 6.61, p = 0.013). Post-hoc comparisons showed that in the Placebo-IF group there was an increase of the percentage of strong pressures in the final session (64.93 ± 7.82%) compared to baseline (49.07 ± 1.13%, p = 0.009), whereas in the Placebo-EF group the percentage of strong pressures was lower in the final session (24.0 ± 8.4%) compared to baseline (48.62 ± 1.22%, p = 0.023). No difference across sessions was found in the Control-IF group (p = 0.785) and in the Control-EF group (p = 0.947). Moreover, the factor Group was significant in the final session (F(3,50) = 5.16, p = 0.003). As revealed by the multiple comparisons, in the final session the Placebo-IF group had higher percentage of strong pressures than the Placebo-EF group (p = 0.003) and, with a tendency, than the Control-EF group (33.54 ± 8.4%, p = 0.052) (Fig. 3C).

### Subjective data

#### Feeling of force

**Figure 4:**
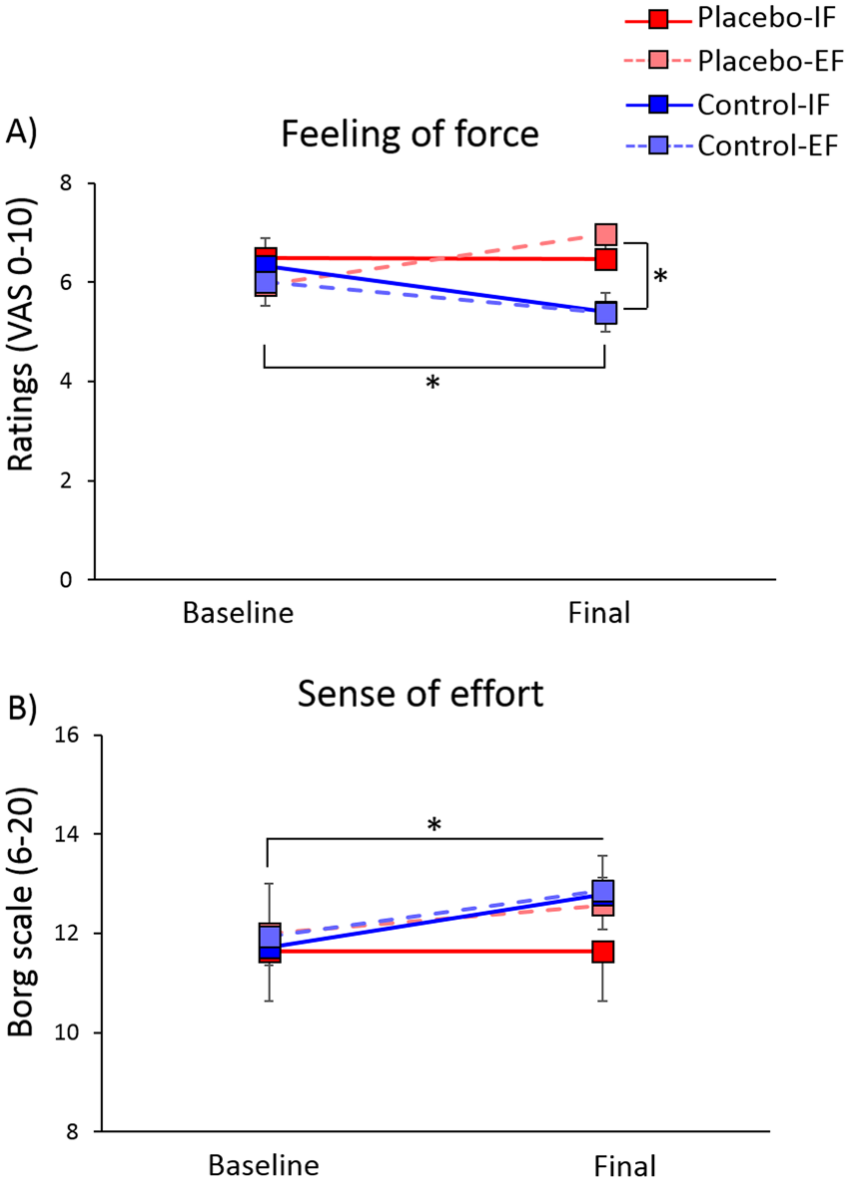
Mean values ± SEM of the subjective variables. Perception of force (**A**) and sense of effort (**B**) in the placebo groups (red squares) and in the control groups (blue squares), with internal focus of attention (IF, dark coloured squares) and with external focus of attention (EF, light coloured squares), in the baseline and final sessions. The Placebo procedure induced higher perception of force in the final session compared to the control procedure. In the control procedure there was a decrease in the perception of force across sessions. The sense of effort generally increased across sessions, but not specifically for single groups. *Significant values, p < 0.050.

Analysis of the feeling of force revealed a significant effect of Procedure (F(1,55) = 4.35, p = 0.042), due to higher feeling of force in the placebo procedure (6.47 ± 0.23) compared to the control procedure (5.79 ± 0.24). The interaction Session × Procedure was also significant (F(1,55) = 9.83, p = 0.003). Post-hoc comparisons showed that in the control procedure the feeling of force was lower in the final session (5.4 ± 0.28) compared to baseline (6.17 ± 0.24, p = 0.001). Moreover, a difference was found between the two procedures in the final session (p = 0.001) with higher values in the placebo procedure (6.7 ± 0.27) compared to the control procedure (5.4 ± 0.28) (Fig. 4A).

#### Sense of effort

Box-Cox transformation^43^ was applied also to the sense of effort scores, since they were not normally distributed (Shapiro-Wilk, p < 0.050). Analysis revealed only a significant effect of Session (F(1,52) = 8.98, p = 0.004), due to higher sense of effort in the final session (12.4 ± 0.21) compared to baseline (11.8 ± 0.19) (Fig. 4B).

### EMG data

**Figure 5:**
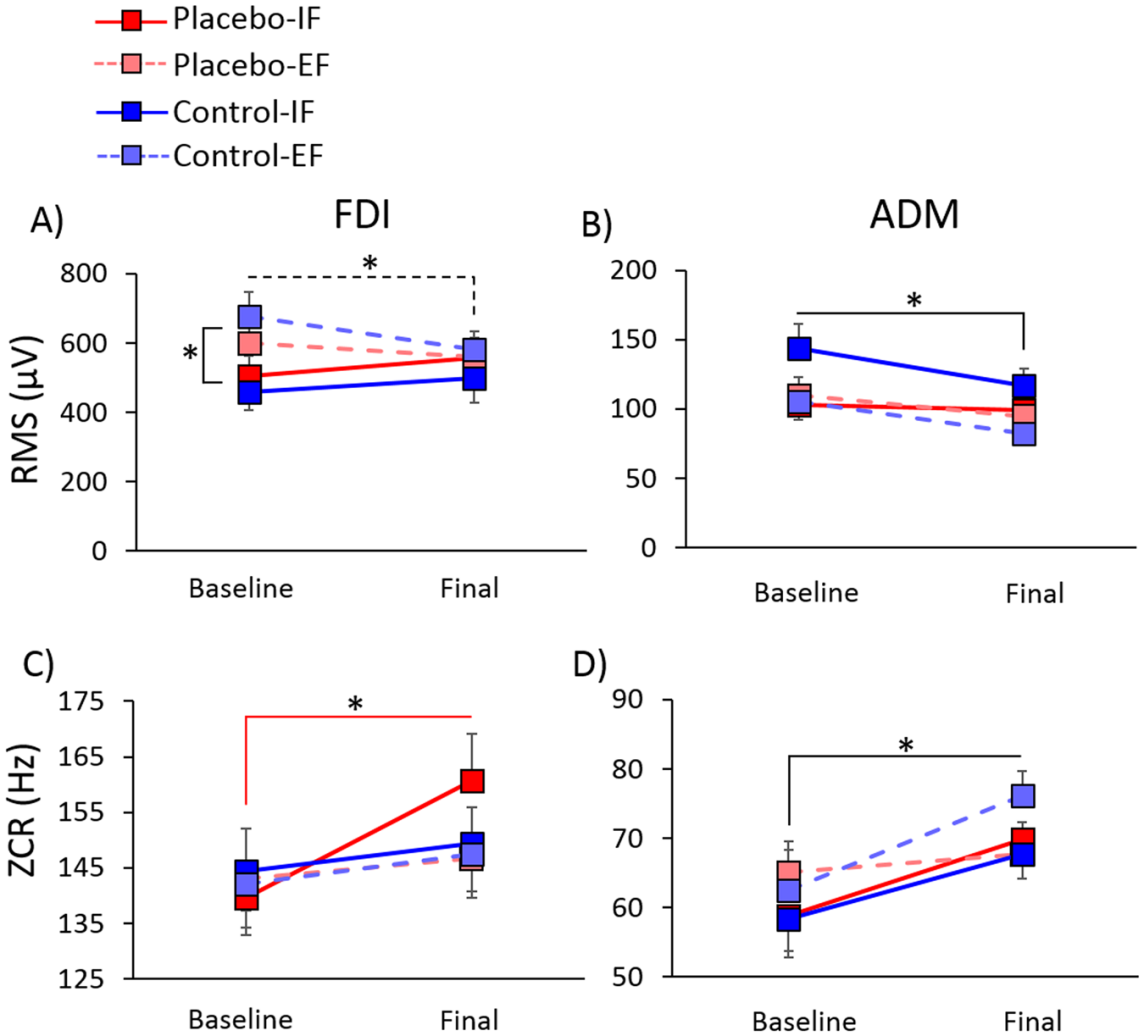
Mean ± SE of the EMG indices. Root mean squared (RMS) decreased in the FDI in the external focus of attention (**A**) and generally decreased in the ADM muscle (**B**). Zero cross rate (ZCR) of the FDI (**C**) significantly increased only in the Placebo-IF group (red line), whereas a general increase was found in the ADM (**D**). *Significant values, p < 0.050.

Analysis of the RMS of the FDI muscle revealed a significant interaction Session × Focus of attention (F(1,48) = 6.28, p = 0.016). Post-hoc comparisons showed that RMS values in the baseline session were higher for the external (639.1 ± 45.27 μV) compared to the internal focus of attention (481.7 ± 40.03 μV, p = 0.013). Moreover, in the case of external focus of attention RMS values were higher in the baseline compared to the final session (570.14 ± 35.93 μV, p = 0.032) (Fig. 5A).

Analysis of the RMS of the ADM muscle revealed only a significant effect of Session (F(1,48) = 7.91, p = 0.007), due to higher values in the baseline (115.4 ± 6.7 μV) compared to the final session (98.04 ± 4.7 μV) (Fig. 5B).

Analysis of the ZCR of the FDI muscle revealed a significant effect of Session (F(1,48) = 15.0, p < 0.001), due to lower values in the baseline (142.3 ± 3.5 Hz) compared to the final session (151.03 ± 3.7 Hz). The interaction Session × Procedure × Focus of attention was nearly significant (F(1,48) = 3.95, p = 0.053). Post-hoc comparisons showed that in the Placebo-IF group ZCR values were higher in the final session (160.62 ± 8.52 Hz) compared to baseline (139.58 ± 6.64 Hz, p = 0.012) (Fig. 5C). No difference between sessions was found in the Placebo-EF group (p = 0.110), in the Control-IF (p = 0.405) group and in the Control-EF group (p = 0.102). The factor Group was not significant neither in the baseline (p = 0.966) nor in the final session (p = 0.494).

Analysis of the ZCR of the ADM muscle revealed only a significant effect of Session (F(1,48) = 10.32, p = 0.002), due to lower values in the baseline (61.23 ± 2.74 Hz) than in the final session (70.37 ± 1.88 Hz) (Fig. 5D).

## Discussion

The results of this study show for the first time that the placebo effect in the motor domain can be influenced by the direction of the focus of attention. A placebo procedure associated with an internal focus of attention resulted in an increase of force production, as shown in the Placebo-IF group. Conversely, the same placebo procedure associated with an external focus of attention induced a reduction of force, but only in the percentage of strong pressures, as shown in the Placebo-EF group. This pattern of results suggests that the placebo effect in the motor domain can be enhanced or reduced by the internal or external focus of attention, respectively, and that the focus of attention can have a different impact on motor performance if a placebo procedure is adopted. Although a similar pattern could be qualitatively observed in all the three behavioural indexes of motor performance (Force_peak_, Normalized Force_peak_, percentage of strong pressures), a marked difference between groups was obtained especially for the percentage of strong pressures. This index represents how many times the subject pressed the piston above a certain value and therefore it gives a fine-tuned picture of the consistency of behaviour. The fact that the main differences between groups emerged more clearly for this index than for the others suggests that the placebo procedure in interaction with the focus of attention impacts more on the way in which individuals persist in their motor performance rather than on the overall amount of force.

The lack of difference between the two control groups (IF and EF), as well as the lack difference between the internal and external focus of attention in the baseline session suggests that the focus of attention *per se* did not modulate force production in our study. This finding is in contrast with the literature on the effects of the focus of attention on motor performance and could be explained by the nature of the task. In our study the task required force exerted by a single finger, whereas most of the studies on the focus of attention considered more challenging motor tasks^44,45^. Different lines of evidence converge in indicating that the EF leads to better motor performance than the IF^19,20^. The EF is characterized by the fact that attention is directed to the goal of the action, while in the internal focus attention is directed toward a specific body district involved in the movement^35^. According to the *constrained action theory*^46^, the EF stimulates automatic processes, that enhance the effectiveness and the efficiency of motor performance, whereas the IF creates some noise in the motor system, that negatively influences the quality of motor performance. In relation to this, the EF has beneficial effects not only on the motor outcome but also on the neuromuscular patterns of movement execution, as evidenced by reduced EMG activity^21,22,47,48^.

In our study, we found higher force levels in the final compared to the baseline session when attention was directed to the body during the placebo procedure. This finding suggests that the placebo effect (which induces itself enhancement of force^16^) may benefit from an IF. This hypothesis is in line with a previous study on pain showing that when subjects focused their attention to the somatic experience after the ingestion of a placebo capsule, the placebo responses were enhanced, whereas if attention was not directed towards the somatic experience, the placebo effect was weak^18^. Attention could be the cognitive mechanism at the basis of this modulation^49^. Following the hypothesis given by Geers, *et al*.^18^, it could be suggested that the expectation induced through the placebo procedure could have influenced motor performance by biasing attention towards the bodily movements. Having applied a treatment on the muscle involved in the task, together with information about its powerful effects on force production, could have induced participants to pay attention for the effects to be perceived. This could have favored the motor performance in the group of subjects whose attention induced by the placebo procedure was located in the same direction as the focus of attention induced by the explicit verbal information (i.e., the Placebo-IF). In other words, the Placebo-IF group had the attention directed to the movements of the finger that was the same body district on which the placebo treatment was applied. This congruency could have induced a better motor outcome. In the Placebo-EF group, instead, the focus of attention was directed to the piston, but the placebo treatment was applied to the finger performing the movements. Hence, in this group the incongruent direction of attention could have hindered the motor outcome.

It could be argued that in the final session, when the cursor amplification introduced in the manipulation session was removed, participants of the Placebo-IF group intentionally increased their level of force in order to maintain the same feedback as in the conditioning phase. However, the fact that only the Placebo-IF group was stronger, and not the Placebo-EF group, despite both groups having been conditioned, suggests that the force enhancement of the Placebo-IF group cannot be attributable exclusively to the intention to be consistent with the conditioning. Moreover, participants of the Placebo-IF group achieved higher levels of force in the final session without increasing their sense of effort, supporting a role of the placebo effect on the force enhancement.

An alternative explanation for the behavioral effect observed in the Placebo-IF group could be related to the higher proneness to respond energetically in the final session because of lower force impressed during the manipulation session. However, the fact that the level of force in the manipulation session was comparable between groups (see Supplementary information) rules out also this possibility.

Recent studies hint at particular situations in which the IF represents an advantage for motor performance. For instance, the IF enhances motor learning in healthy participants who prefer kinesthetic motor imagery, whereas the EF has positive effects in those who prefer visual motor imagery^50^. This suggests that inter-individual differences in the motor imagery abilities may modulate the effects of the focus of attention on the motor outcome. The same finding has been described in patients with stroke: those with preference for kinesthetic motor imagery present with better movement accuracy under the IF^23^. This is in line also with another study showing that stroke patients prefer adopting an IF^51^. Hence, as also shown by our findings, the EF may not always be the best strategy for motor control.

It should be noted that, differently from most of the studies on the focus of attention^19^, in our study we used a visual feedback, in addition to the verbal instructions, to guide participants either to the IF or to the EF. This methodological choice was due to the need of conditioning participants of the placebo groups about the effects of TENS in increasing force. Thanks to the surreptitious amplification of the visual feedback, indeed, we could reinforce subjects’ belief about the effects of TENS^16,33^. It is known that a combination of conditioning and verbal suggestion is more powerful than verbal suggestion alone in inducing placebo effect^37^. With this aim in mind, we tried to limit as much as possible the potential distracting effect of the visual feedback *per se* on the capacity to maintain the requested direction of the attentional focus (either on the finger or on the piston), by manipulating the context in which the feedback was anchored. Specifically, the context of the visual feedback was congruent with the direction of the attentional focus, and therefore the cursor was anchored on the finger in the case of the IF and on the piston in the case of the EF. In both cases, however, the absolute position of the visual feedback on the screen (computed as distance from the boarder of the monitor) was the same, in order to avoid any effect due to different spatial orientation of visual attention. Moreover, in both cases, the visual feedback could be considered as a translation on the PC monitor of the direction of the focus of attention induced through the verbal instructions. Specifically, in the case of the EF participants looked at an image of the piston, not at the actual piston and in the case of the IF participants looked at an image of the finger, not at the actual finger. Hence, in the two conditions (EF and IF) the visual feedback had exactly the same function.

It could be argued whether the condition we called “internal focus of attention” could be defined as “internal” even if an “external” visual feedback was adopted. The choice of defining this condition as internal is strictly related to the type of verbal instructions (i.e., to focus the attention on the movements of the fingers). Recent evidence shows that the visual information (e.g., a screen) can be used as a tool to guide the participant’s attentional focus (internal and external) during the execution of motor task^23,24,50^. Nonetheless, the ability to direct the attentional focus is not influenced by the visual feedback *per se* but rather by the content of the verbal instructions^19,52,53^. This suggests that what matters in the attentional focus is the verbal instruction and the visual information *per se* does not undermine the ability to direct the attentional focus^52,53^. The fact that participants in our study declared to have been capable to maintain the focus of attention throughout the procedure is in line with previous studies^23,29,36,54–65^ and suggests that the visual feedback did not distract them from the focus. The use of a subjective method, like a VAS, to measure the participants’ focus of attention could be considered as a limitation, due to the retrospective nature of the assessment and the plausibility that participants rated the duration of the focus in order to please the experimenter’s request^66^. Nonetheless, this approach represents a common methodological choice and therefore it allows comparisons with other studies^23,29,36,54–65^. Moreover, the fact that in our study the mean VAS scores ranged from 4.9 to 7.1, suggests that participants did not always give the highest score possible (i.e., 10), thus ruling out a mere effect of compliance. Finally, to ensure that participants consistently maintained their attentional focus, the experimenter repeatedly encouraged them throughout the task to direct the attention in the external or internal direction. Future investigations adopting more objective measures (e.g. using an action sport camera or a head mounted eye tracker)^67^, may help improving the validity and the reliability of the assessment.

Subjective data revealed that the participants of the Placebo-EF group had positive expectations of improvement compared to the control groups. Nonetheless, the percentage of strong pressures in this group was reduced in the final session. These findings hint dissociation between the subjective and objective components of the placebo effect. A similar dissociation was found also in a recent study by Schwarz and Buchel^68^. In a cognitive task, they found that participants perceived the effects of a treatment (actually inert) in improving performance, despite the lack of effect in the objective measure of actual performance^68^. In our case, we could speculate that, as discussed above, in Placebo-EF the mismatch between the location of the placebo treatment (on the finger) and the direction of the focus of attention (on the piston) could have impaired actual motor performance, despite positive expectations. Previous studies showed that the direction of the attentional focus can interact also with psychological variables, such as self-determination and self-efficacy^62,69^. In particular, the EF is associated with the perception of higher levels of auto-efficacy and less difficulty during the execution of a motor task compared with the IF^62,69^. Although we did not assess psychological traits, it is reasonable that the EF induced participants to expect to be stronger because of a higher sense of self-efficacy, without this necessarily turning into an improvement of motor performance. Future investigations showed better clarify this dissociation.

With regards to the electromyographic activity, we found a peculiar pattern of results in the Placebo-IF group, that was different with respect to the other groups. Typically, muscular contraction is controlled with two different neural mechanisms: firing rate coding and motor unit recruitment^70,71^. Firing rate coding indicates the motor unit firing frequency, whereas motor unit recruitment represents the total number and type of motor units activated^72^. These two mechanisms can be measured with specific EMG parameters, like RMS which is related to the firing rate and ZCR (as a representative of EMG power spectrum) which is correlated to motor unit recruitment^71,73^. In our study, the electromyographic activity measured in terms of RMS decreased from the baseline to the final session, both for the FDI and the ADM muscles. In the case of the FDI muscle, the RMS decrease was specific for the external focus of attention. In virtue of the relationship between the firing rate and the RMS^74^, this result suggests that the firing rate decreased across the experimental procedure. Different studies showed that there is a relationship between force and RMS, by which higher force levels correspond to higher RMS values^75,76^. The general decrease of the RMS in our study may hint at a decrease of force across sessions, despite the latter being significant only for the Placebo-EF group in terms of reduction of percentage of strong pressures. The fact that RMS decreased, despite the increase of force displayed by the Placebo-IF group hints at a peculiar neuromuscular strategy in this group that could be explained by taking into account the complete picture of EMG activation, including ZCR. This index is a good predictor of muscle damage and fatigue after endurance^77^. Specifically, shifts toward low frequencies in this parameter are indicative of muscle fatigue^39–41^. The fact that ZCR in our study did not decrease indicates that the motor task did not induce muscular fatigue. It should also be noted that ZCR has a linear relationship with motor unit action potentials, by which higher ZCR is indicative of enhancement in muscle recruitment^41,73^. In our study, the Placebo-IF group had an increase of ZCR in the final compared to the baseline session, suggesting that the higher force levels shown by this group could have been obtained by enhancing muscle units recruitment without changing firing rate. This neuromuscular strategy could have resulted in persistence of force levels without muscle fatigue. In terms of muscle fiber recruitment between the two sessions, the result obtained in the ADM muscle resembles those of the FDI. This could hint at a spreading of the same neuromuscular strategy from the FDI to the ADM, although in the case of the ADM this was not specific for the Placebo-IF group.

The use of cognitive strategies to facilitate motor performance is an increasing practice in sport and physical exercise. The focus of attention^34^ and the placebo effect^4^ represent, among others, two suitable methods to improve the motor outcome. In this explorative study, we combined for the first time these approaches and demonstrated that the placebo effect in the motor domain may benefit from an IF. Future research along this line could help to elucidate the mechanisms at the bases of these effects, as well as the best procedure to induce a favourable outcome not only in athletes but also in patients suffering from different types of motor deficits.

## Electronic supplementary material

Supplementary information
