# Supplementary material for: The placebo effect in the motor domain is differently modulated by the external and internal focus of attention
Source: Sci Rep. 2018 Aug 16;8:12296. doi: 10.1038/s41598-018-30228-9 (PMC6095847; doi:10.1038/s41598-018-30228-9)
Supplement: Supplementary file 1 — Supplementary information [file 41598_2018_30228_MOESM1_ESM.docx]

**Supplementary information**

**The placebo effect in the motor domain is differently modulated by the external and internal focus of attention**

Rossettini Giacomo^1,2#^, Emadi Andani Mehran^1#^, Dalla Negra Francesco^1^, Testa Marco^2^, Tinazzi Michele^1^, Fiorio Mirta^1*^

^1^ Department of Neurosciences, Biomedicine and Movement Sciences, University of Verona, Verona, Italy.

^2^ Department of Neuroscience, Rehabilitation, Ophthalmology, Genetics, Maternal and Child Health, University of Genova, Campus of Savona, Italy.

**Computation of the sample size**

Computation of the sample size was performed with G-Power 3.1^1^, considering F tests within-between interaction with four groups and two sessions (baseline and final). To determine the effect size f, we proceeded by estimating it a priori, based on a given partial eta square. Assuming a priori a medium partial eta square of 0.05^2^ the effect size f is equal to 0.2294. Given this effect size, α error probability of 0.05, power (1-β error probability) of 0.90, correlation among repeated measures of 0.6 and nonsphericity correction ε of 1, the resulting total sample size is 60.

**References**

1. Faul, F., Erdfelder, E., Lang, A.G. & Buchner, A. G*Power 3: a flexible statistical power analysis program for the social, behavioral, and biomedical sciences. *Behav. Res. Methods*, **39**(2), 175-91, (2007).
2. Bakeman, R. Recommended effect size statistics for repeated measures designs. *Behav. Res. Methods* **37**, 379-384, (2005).

**Table S1. Number of outliers in each variable and group**

| **Variable** | **Placebo-IF** | **Placebo-EF** | **Control-IF** | **Control-EF** |
| --- | --- | --- | --- | --- |
| Force_peak_ | 1 | 0 | 1 | 1 |
| Normalized Force_peak_ | 1 | 1 | 1 | 1 |
| Percentage of strong pressures | 0 | 2 | 2 | 2 |
| Feeling of force | 0 | 0 | 1 | 0 |
| Sense of effort | 1 | 1 | 1 | 1 |
| RMS FDI and ADM | 2 | 2 | 3 | 1 |
| ZCR FDI and ADM | 1 | 3 | 2 | 2 |

**Manipulation session**

The conditioning procedure introduced in the manipulation session could have induced participants of the placebo groups to reach higher levels of the visual feedback with a smaller force. Hence, the behavioral data of the manipulation were further analyzed to check whether the four groups had the same amount of force in this session.

*Statistical analysis*

One-way ANOVA with the factor Group (Placebo-IF, Placebo-EF, Control-IF, Control-EF) was carried out to analyze the difference of force between groups in the manipulation session. Moreover, paired-sample t-tests were conducted to compare the manipulation session with the baseline and final sessions in each group separately. Bonferroni correction for multiple comparisons was applied where necessary. The level of significance was set at p < 0.050.

*Results*

As revealed by the statistical analysis (one-way ANOVA), the factor Group was not significant (Force_peak_: p = 0.433, Normalized Force_peak_: p = 0.126; percentage of strong pressures: p = 0.177), indicating that the four groups did not have different force levels in the manipulation session. Moreover, within-group comparisons (paired-sample t-tests) revealed that the force during the manipulation session was not different from the baseline and final sessions (see Table S2 for all the comparisons). These results demonstrate that the conditioning procedure did not induce a reduction of force in the placebo groups during the manipulation session.

**Table S2. Bonferroni-corrected p values of the paired-sample t-tests in each group**

|  | **Manipulation vs Baseline** | | | **Manipulation vs Final** | | |
| --- | --- | --- | --- | --- | --- | --- |
|  | **Force_peak_** | **Normalized Force_peak_** | **Percentage of strong pressure** | **Force_peak_** | **Normalized Force_peak_** | **Percentage of strong pressure** |
| **Placebo-IF** | p = 1 | p = 0.297 | p = 0.354 | p = 0.180 | p = 0.321 | p = 0.453 |
| **Placebo-EF** | p = 0.267 | p = 0.615 | p = 1 | p = 1 | p = 1 | p = 0.762 |
| **Control-IF** | p = 0.729 | p = 0.618 | p = 1 | p = 1 | p = 1 | p = 1 |
| **Control-EF** | p = 0.438 | p = 0.804 | p = 1 | p = 1 | p = 1 | p = 1 |

**Table S3. Sensitivity test to compare the statistical significance with and without outliers**

|  | **Force_peak_** | **Normalized Force_peak_** | **Percentage of strong pressures** | **Feeling of force** | **Sense of effort** | **RMS FDI and ADM** | **ZCR FDI and ADM** |
| --- | --- | --- | --- | --- | --- | --- | --- |
| Session | = | = | = | = | = | = | = |
| Procedure | = | p = 0.063 | = | = | = | = | = |
| Focus of attention | = | = | = | = | = | = | = |
| Session × Procedure | = | = | = | = | = | = | = |
| Session × Focus of attention | = | = | p = 0.054 | = | = | = | = |
| Procedure × Focus of attention | = | = | p = 0.125 | = | = | = | = |
| Session × Procedure × Focus of attention | p = 0.049 | p = 0.087 | p = 0.083 | = | = | = | = |

The sign “=” indicates the same results with and without outliers. Cells in which the p value is reported indicate different results obtained by including the outliers.
